# Benchmarking Cross-Docking Strategies in Kinase Drug Discovery

**DOI:** 10.1021/acs.jcim.4c00905

**Published:** 2024-11-19

**Authors:** David A. Schaller, Clara D. Christ, John D. Chodera, Andrea Volkamer

**Affiliations:** † In Silico Toxicology and Structural Bioinformatics, Institute of Physiology, Charité − Universitätsmedizin Berlin, corporate member of Freie Universität Berlin and Humboldt-Universität zu Berlin, Augustenburger Platz 1, 13353 Berlin, Germany; ‡ Computational and Systems Biology Program, Sloan Kettering Institute, Memorial Sloan Kettering Cancer Center, New York, New York 10065, United States; § Structural Biology & Computational Design, Drug Discovery Sciences, Pharmaceuticals, Bayer AG, 13342 Berlin, Germany; ∥ Data Driven Drug Design, Faculty of Mathematics and Computer Sciences, Saarland University, 66123 Saarbrücken, Germany

## Abstract

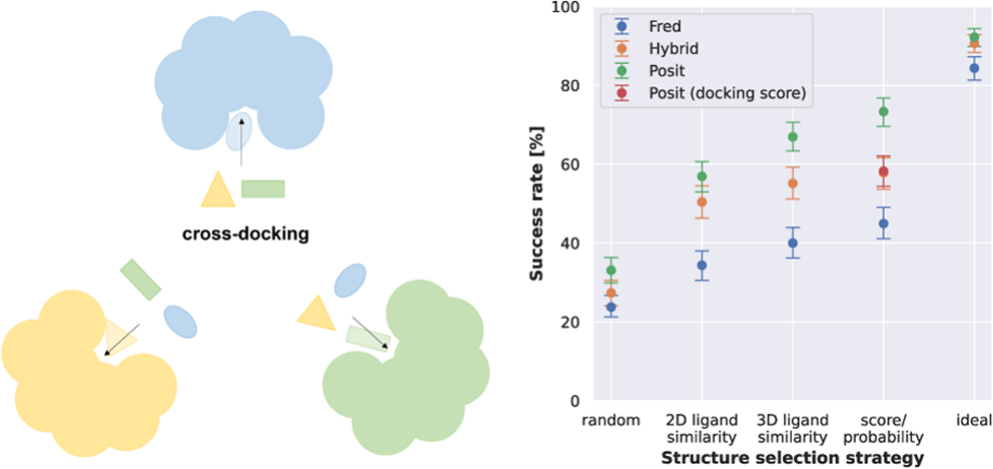

In recent years, machine learning has transformed many aspects of the drug discovery process, including small molecule design, for which the prediction of bioactivity is an integral part. Leveraging structural information about the interactions between a small molecule and its protein target has great potential for downstream machine learning scoring approaches but is fundamentally limited by the accuracy with which protein–ligand complex structures can be predicted in a reliable and automated fashion. With the goal of finding practical approaches to generating useful kinase-inhibitor complex geometries for downstream machine learning scoring approaches, we present a kinase-centric docking benchmark assessing the performance of different classes of docking and pose selection strategies to assess how well experimentally observed binding modes are recapitulated in a realistic cross-docking scenario. The assembled benchmark data set focuses on the well-studied protein kinase family and comprises a subset of 589 protein structures cocrystallized with 423 ATP-competitive ligands. We find that the docking methods biased by the cocrystallized ligand, utilizing shape overlap with or without maximum common substructure matching, are more successful in recovering binding poses than standard physics-based docking alone. Also, docking into multiple structures significantly increases the chance of generating a low root-mean-square deviation (RMSD) docking pose. Docking utilizing an approach that combines all three methods (Posit) into structures with the most similar cocrystallized ligands according to the maximum common substructure (MCS) proved to be the most efficient way to reproduce binding poses, achieving a success rate of 70.4% across all included systems. The studied docking and pose selection strategies, which utilize the OpenEye Toolkits, were implemented into pipelines of the KinoML framework, allowing automated and reliable protein–ligand complex generation for future downstream machine learning tasks. Although focused on protein kinases, we believe that the general findings can also be transferred to other protein families.

## Introduction

Machine learning (ML) has found its way into many aspects of the drug discovery process.^1^ Reliably predicting the bioactivity of small molecules binding to disease-relevant targets has the potential to accelerate ligand design and to reduce costs, especially in the early drug design phases.^2^ While ligand-based ML models, which use the ligand chemical structure, are now widely used in drug discovery,^3−5^ structural information, where protein–ligand complex structures are encoded, in principle, contains more information that can be used to improve affinity predictions. While it has so far proven difficult for structure-based methods to demonstrate practical improvements over ligand-based methods,^6−8^ the highly limited number of experimentally resolved protein–ligand complexes (compared to relatively more abundant protein–ligand affinity data) raised concerns about the generalizability of such models to a chemical space and protein targets not used in the training process.^7,9^ Several studies have assessed the ability of ML models utilizing protein–ligand complex structures to predict binding affinity data.^7,10,11^ However, applying such approaches prospectively requires a reliable framework for generating experimentally unresolved protein–ligand complexes capable of capturing the relevant ligand binding mode(s) with sufficient accuracy. A number of new ML-based frameworks have been developed for predicting protein–ligand complexes that show potential^12−14^ but have so far proven unable to rival traditional docking methods.^15^ The recently released AlphaFold 3 model shows impressive performance in predicting protein–ligand complexes, but the currently limited accessibility prevents its comparison to other approaches.^16^ More traditional docking approaches sample docking poses inside a defined binding pocket of protein structures and select poses with physics-based scoring functions.^17^

For structure-based machine learning methods to provide more utility than ligand-based methods alone, protein–ligand complex poses must be predicted with sufficient accuracy to both (1) allow poses and interactions to be determined prospectively for molecules that have not yet been synthesized and (2) allow ligand poses to be inferred if experimental structures are unavailable for protein–ligand complexes for which affinity data are available and would be useful in model training. While redocking benchmarks have proven useful in estimating the performance of docking tools to reproduce experimentally resolved X-ray structures,^18,19^ the results cannot easily be translated into a prospective setting in which small molecules are docked into protein structures with binding pockets fitted to other cocrystallized ligands or without bound ligand. Furthermore, the strategy to select an experimentally resolved protein structure for docking a particular small molecule heavily influences the docking performance and is consequently equally important to assess. A few studies have been performed in this direction, identifying the 3D similarity of the cocrystallized ligand and the binding site size as important parameters to select a suitable protein structure for docking.^20−22^ However, these benchmark studies did not cover 2D similarity and were not embedded in fully automated pipelines, which is critical for high throughput complex generation and subsequent machine learning experiments on the available binding affinity data.

The OpenKinome initiative represents a collaborative effort to build the infrastructure for controlled computational experiments toward structure-informed machine learning for bioactivity predictions of small molecules. For the development process, the focus has been put on protein kinases, an important protein family for developing anticancer drugs.^23^ Protein kinases are well studied, resulting in abundant data available for both ligand bioactivity^24,25^ and experimentally resolved X-ray structures,^26,27^ though with orders of magnitude more bioactivity measurements available than experimental structures. Here, we present the results of a cross-docking benchmark for docking pipelines implemented into the KinoML framework. We pay special attention to the conformational heterogeneity of protein kinases and evaluate several strategies to generate and select low RMSD docking poses. The code is made publicly available in the kinase-docking-benchmark repository for the generation and analysis of the docking results, as well as in the KinoML repository for the corresponding docking pipelines.

## Results and Discussion

### Creation of a Thorough Cross-Docking Benchmark Data Set for Protein Kinases

The OpenCADD-KLIFS module^27,29^ was used to generate the cross-docking benchmarking data set for this study (Table 1). The prerequisite for kinase structures to be included in the benchmark data set was having a fully resolved ATP-binding site with a wild-type sequence to exclude any influence of modeling missing residues or mutations. Also, we included only kinases with at least 10 different structures of a kinase in a distinct KLIFS kinase conformation (namely, DFG in/out and αC helix in/out, which correlate with the active and inactive states of a kinase)^30^ with a single cocrystallized ligand in the ATP-binding site. This procedure resulted in the final selection of 589 structures covering 10 distinct kinases and 423 ligands crystallized in a total of four different conformations (Table 1, Figure S1).

**Table 1:** Kinase-Inhibitor Cross-Docking Benchmark Set Includes a Representative Set of Kinases in a Variety of Conformations^a^

organism	kinase name	kinase group	DFG	αC helix	number
human	PIM1	CAMK	in	in	129
human	AurA	other	in	in	95
human	JAK2	TK	in	in	61
human	BTK	TK	in	out	56
mouse	PKACα	AGC	in	in	54
human	IRAK4	TKL	in	in	45
human	MAP2K1	STE	in	out	42
human	MST3	STE	in	in	29
human	AurA	other	out-like	in	24
human	TRKA	TK	out	in	22
human	BRAF	TKL	in	out	21
human	BRAF	TKL	out	in	11

a A benchmark set of 589 kinase inhibitor structures (representing 10 kinases in a variety of conformations) was constructed to ensure that ligands should be able to be robustly placed within the correct binding pose. We selected kinases with cocrystallized ligands in the ATP-binding site for which at least 10 structures were observed in the same DFG and αC helix conformation and for which all 85 KLIFS residues contacting the ATP-binding site were both crystallographically resolved and contained wild-type residues only (benchmark set provided in the Supporting Information). The 85 KLIFS residues are key residues for ligands binding to the orthosteric binding site of protein kinases, including all key residues involved in ATP binding, as well as important structural regions, i.e., DFG-motif, activation loop, and αC helix.^28^

Protein kinases are a conformationally heterogeneous protein family.^27,31^ ATP-competitive inhibitors commonly prefer binding to a single kinase conformation but have also been found to bind to different (or multiple) kinase conformations depending on construct length, phosphorylation, or mutations (Table 2).^32−34^

**Table 2:** Examples of Orthosteric Protein Kinase Ligands That Bind to Different Kinase Conformations^a^

expo ID	kinase	DFG/αC helix conformation–PDB entry	alteration
ATP	CAMK1	in/in - 4FG7, in/out - 4FG9, out-like/out - 4FG8	construct length
OWQ	MAPK6	in/in - 6YKY (chain B), out-like/in - 6YKY (chain A), out-like/out - 6YKY (chain D)	none
OWX	MAPK6	in/in - 6YLC (chain B), out-like/in - 6YLC (chain A), out-like/out - 6YLC (chain D)	none
ACP	AurA	in/in - 6CPF, out-like/in - 4C3R, out/in - 6C83	nanobody
ADN	AurA	in/in - 4O0S, out-like/in - 4O0U, out/in - 1MUO	construct length, mutation
F9N	NEK7	in/in - 6S73, in/out - 6S75 (chain B), out-like/out - 6S75 (chain A)	mutation
STU	MAP3K5	in/in - 4BF2 (chain B), in/out - 4BF2 (chain A), out-like/in - 2CLQ (chain B), out-like/out - 2CLQ (chain A)	construct length, mutation
B49	MAK4K1	in/in - 6NFZ, in/out - 6NG0 (chain A), out/out - 6NG0 (chain B)	phosphorylation, mutation

a The KLIFS database was searched for orthosteric ligands binding to the same kinase in at least 3 different conformations. These observed conformations can result from alterations in the crystallized construct, e.g., construct length, cocrystallized nanobodies, mutations, and phosphorylation pattern. However, several ligands bind to different conformations of the same crystallized construct. Here, “Expo ID” refers to the chemical component identifier in the RCSB Ligand Expo (or the Chemical Components Dictionary).

This observation complicates cross-docking studies across multiple kinase conformations, since a good docking score of an inhibitor to a kinase structure in a putative wrong conformation may not necessarily mean a poor performance of the docking algorithm. Instead, this could also indicate that the inhibitor may actually also bind to this conformation but was simply not yet resolved with a corresponding construct, allowing it to crystallize in this conformation. Hence, this cross-docking study was performed only within the respective kinase conformations.

The stringent filtering criteria mentioned above reduce the total number of included structures for this docking benchmark tremendously and exclude apo structures. In addition, this benchmark set is limited to a single protein class, i.e., protein kinases. However, the abundant structural data, in conjunction with its detailed annotation in KLIFS, offers a unique chance to assess the docking performance in a realistic cross-docking scenario on an important drug target class.^27^ Also, we believe that the findings can be transferred to other target classes with less available structural information.

### Molecular Docking Recovers Most of the Benchmark Ligand Poses

In this study, the binding site was defined by the 85 KLIFS residues, which account for the majority of protein–ligand interactions observed in X-ray structures of protein kinases.^28^ Three docking algorithms from the OpenEye Toolkits were employed: Fred, Hybrid, and Posit.^35^**Fred** follows a standard docking approach, where possible conformations of flexible ligands are sampled within a rigid protein binding site and evaluated using a physics-based scoring function. **Hybrid**, in contrast, leverages the shape and electrostatics of a cocrystallized small molecule as a template to guide the placement of new ligands in the binding site, favoring those with similar features to the template. **Posit** automatically chooses the most suitable docking algorithm based on the similarity between the cocrystallized ligand and the small molecule to be docked. For example, if the new ligand differs only slightly from the cocrystallized ligand, e.g., by a single methyl group, the new ligand will be placed via maximum common substructure docking.^36^ Ligands with low similarity will be docked with the standard docking approach Fred. Regardless of which docking algorithm is selected, Posit provides a probability score indicating the likelihood that the generated docking pose is within 2 Å of the experimentally determined binding pose.

**Figure 1:**
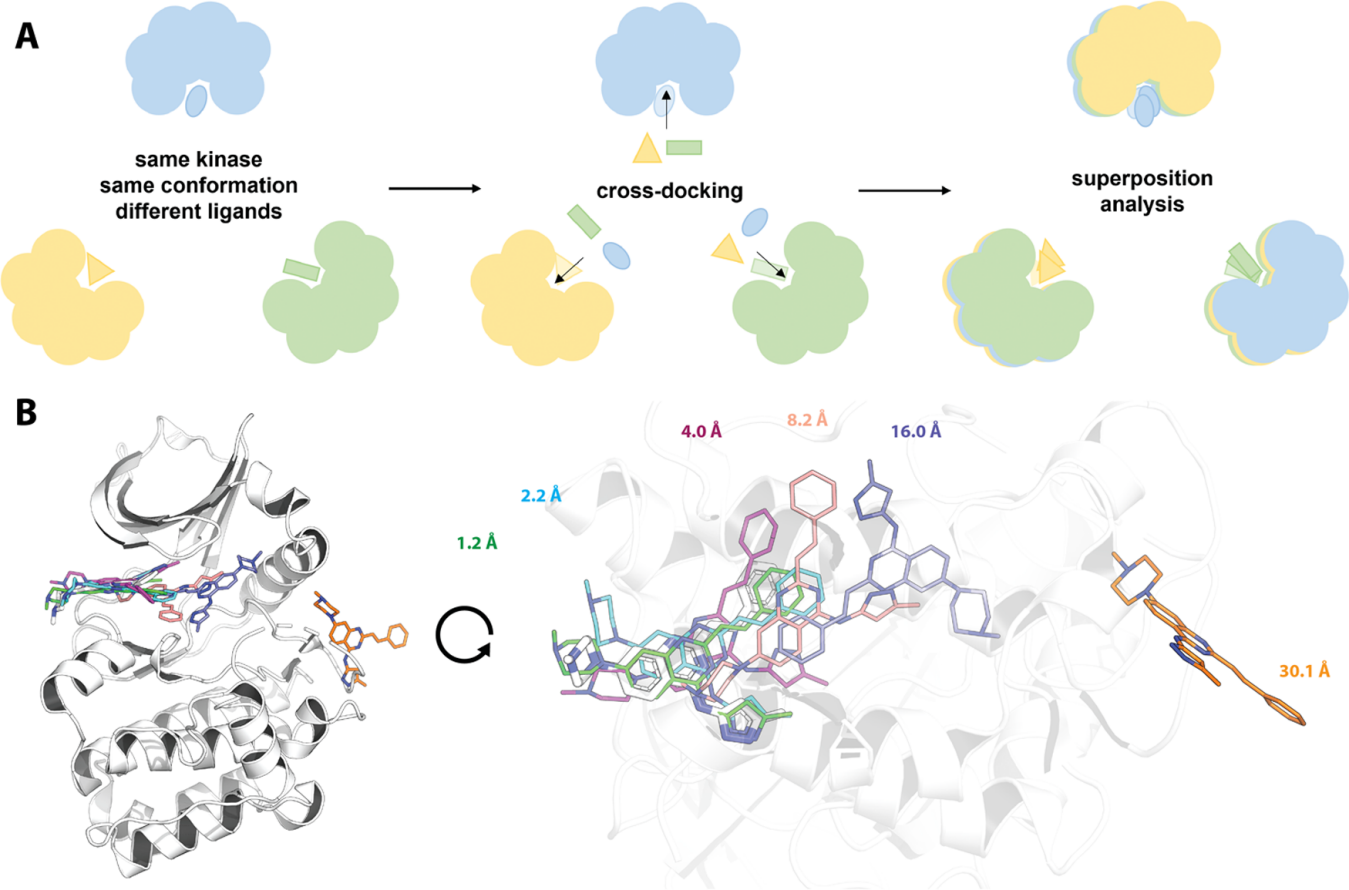
Design of this cross-docking study and visualization of different RMSD results observed in a cross-docking experiment. (A) Experimental design of this cross-docking study from structure selection over the actual cross-docking experiment to the analysis after superposition. In the depicted example, three X-ray structures (blue, yellow, green) of the same kinase resolved in the same conformation cocrystallized with three different ligands are used. Each ligand is docked into the orthosteric binding site of the other two kinase structures. The docked structures are then superimposed to the structure in which the ligand was cocrystallized. Finally, the docking poses are analyzed via calculation of the root-mean-square deviation (RMSD). (B) AurA entry 5ZAN with a cocrystallized ligand is depicted in white; docking poses generated by Fred for highlighted RMSD ranges in different colors. The docking pose with an RMSD below 2 Å recapitulates the binding mode and interactions observed in the X-ray structure. Docking poses with an RMSD up to 4 Å are located in the correct binding site. Docking poses with an RMSD of 8 Å or higher are located in other parts or outside of the binding pocket.

The cross-docking study was performed within structures of the same kinase and conformation (Figure 1A). For example, each of the 11 ligands cocrystallized with human BRAF in the DFG out/αC helix in conformation was docked into the remaining 10 structures of this kinase and conformation it was not cocrystallized with (Table 1). This procedure totals ∼40,000 docking runs per cross-docking experiment, in which each ligand is docked into all other available structures of the kinase in the same conformation.

After docking, postprocessing was performed with functionality from the OpenEye Toolkits^35^ by superimposing the protein, including the docking pose, to the reference PDB structure with which the docked ligand was cocrystallized. Finally, the root-mean-square deviation (RMSD) of heavy atoms between cocrystallized ligand and docking pose was calculated. A docking attempt generating a docking pose with an RMSD of ≤2 Å compared to the reference ligand structure was considered successful, which is a commonly used criterion in docking benchmark experiments.^18−22^ Docking poses with an RMSD of 4 Å usually cover the same binding pocket as the cocrystallized ligand, and docking poses with an RMSD >8 Å cover other parts of the binding pocket or are located outside the actual binding site (Figure 1B).

**Figure 2:**
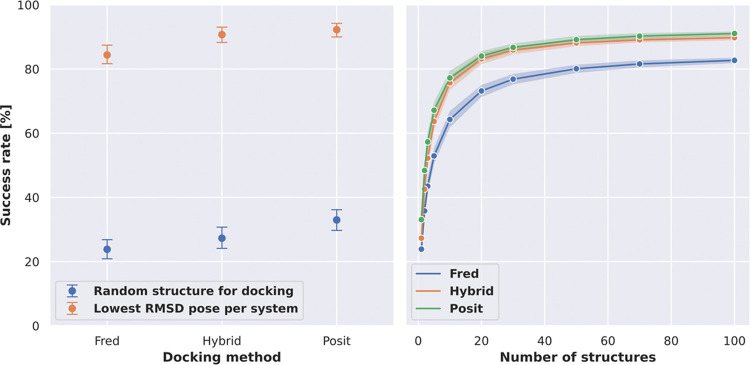
Molecular docking can recapitulate the majority of ligand binding poses, but the success rate heavily depends on the selected kinase structure for docking. **Left**: Benchmark results show that low RMSD docking poses can be generated for the majority of the systems across the 3 docking methods. The selection of the kinase structure for docking has a great impact on the success rate (pose ≤2 Å). **Right**: Success rates to generate docking poses ≤2 Å depend on the number of kinase structures used during docking. Observed differences between Hybrid and Posit are not statistically significant. Reported success rates and confidence intervals were estimated by using bootstrapping. Confidence intervals are depicted as whiskers on the left plot and as filled bright areas surrounding the solid lines on the right plot.

The RMSD analysis revealed that Posit was the most successful docking method, achieving a mean bootstrapped success rate of 33.0% when picking a random kinase structure of the same conformation for docking and 92.2% when considering only the lowest RMSD pose per system to reproduce (Figure 2, left, Figure S2). Physics-based docking alone (Fred) was the least successful method, reproducing the binding pose for only 23.8% of systems when randomly selecting a kinase structure for docking and 84.3% of systems in the case of the lowest RMSD pose generated per system. These results highlight that the choice of a protein structure for docking a particular ligand has a dramatic impact on the performance, which was likewise shown by other studies before.^20−22^ Additionally, the better performance of docking algorithms biased by a cocrystallized ligand is consistent with previous cross-docking benchmarks.^20,21^

### How Can the Chance Be Increased to Generate and Select a Low RMSD Docking Pose?

The success rates differ strongly when analyzing docking poses generated from docking into a randomly selected kinase structure compared with the lowest RMSD docking poses per system (Figure 2 left). Hence, we were interested in identifying strategies to increase the likelihood of generating and selecting low RMSD docking poses.

Due to the design of the benchmark data set, we could simulate the success rate of docking ligands into a different number of available X-ray structures and analyze the effect on generating a low RMSD docking pose. Therefore, the generated docking poses were bootstrapped to select docking poses for a defined number of randomly selected protein kinase structures. Increasing the number of protein structures used for docking steadily increased the chance to generate a low RMSD docking pose (Figure 2, right). Posit and Hybrid methods required 20 structures to surpass a success rate of 80%, and Fred required 50 structures.

We also tested the effect of generating 5 instead of 1 pose per docking attempt. Generating multiple poses increased the success rate to generate a low RMSD pose when picking a random structure for docking. However, the success rate did not improve significantly when analyzing the lowest RMSD pose generated per system (Figure S3). Adding the results for docking into multiple structures (Figure 2), one can conclude that docking into multiple kinase structures is more successful than generating multiple docking poses for a single kinase structure.

**Figure 3:**
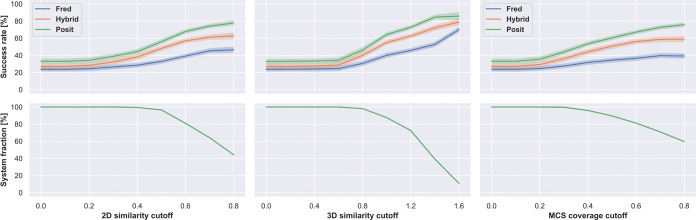
Docking success rates improve when docking into structures with more similar cocrystallized ligands. The 2D similarity of ligands was assessed using Morgan fingerprints via Dice similarity implemented in RDKit. The MCS coverage was calculated using the RDKit library by dividing the number of MCS atoms between the two compounds by the number of heavy atoms in the ligand being docked. The 3D similarity was determined using an overlay of shape and electrostatics via Tanimoto similarity implemented in OpenEye Toolkits.^35^ Reported success rates and confidence intervals were estimated using bootstrapping.

Previous studies have found a positive effect of docking into structures with similar cocrystallized ligands.^20,21^ We could observe a similar behavior with regard to 2D similarity using Morgan fingerprints, 3D similarity estimated with shape and electrostatic criteria, and maximum common substructure (MCS) coverage (Figure 3). However, restricting docking runs to structures with more similar cocrystallized ligands led to the exclusion of many systems for which no cocrystallized ligand was available, meeting the required similarity threshold. For example, applying a 2D similarity cutoff of 0.8 increased the success rate for Posit from 33.0% to almost 80%. However, at this cutoff, only 40% of the systems had a corresponding kinase structure available. In contrast, an MCS coverage cutoff of 0.8 retains 60% of the benchmark systems and also increases the success rate to almost 80%.

Given the increased availability of computational resources, it would be also a viable strategy to dock into all available kinase structures and afterward restrict the analysis to docking poses with sufficiently good docking scores. In this study, we analyzed the Chemgauss4 docking score for all three docking methods and additionally the Posit probability for the Posit method. The Posit probability estimates the likelihood of generating a low RMSD pose based on the 2D and 3D similarity of the ligand to dock and the cocrystallized small molecule (see the documentation for further details). The docking score was normalized by the heavy atom count of the docked molecule to account for the correlation between the docking score and molecular weight.^37^

**Figure 4:**
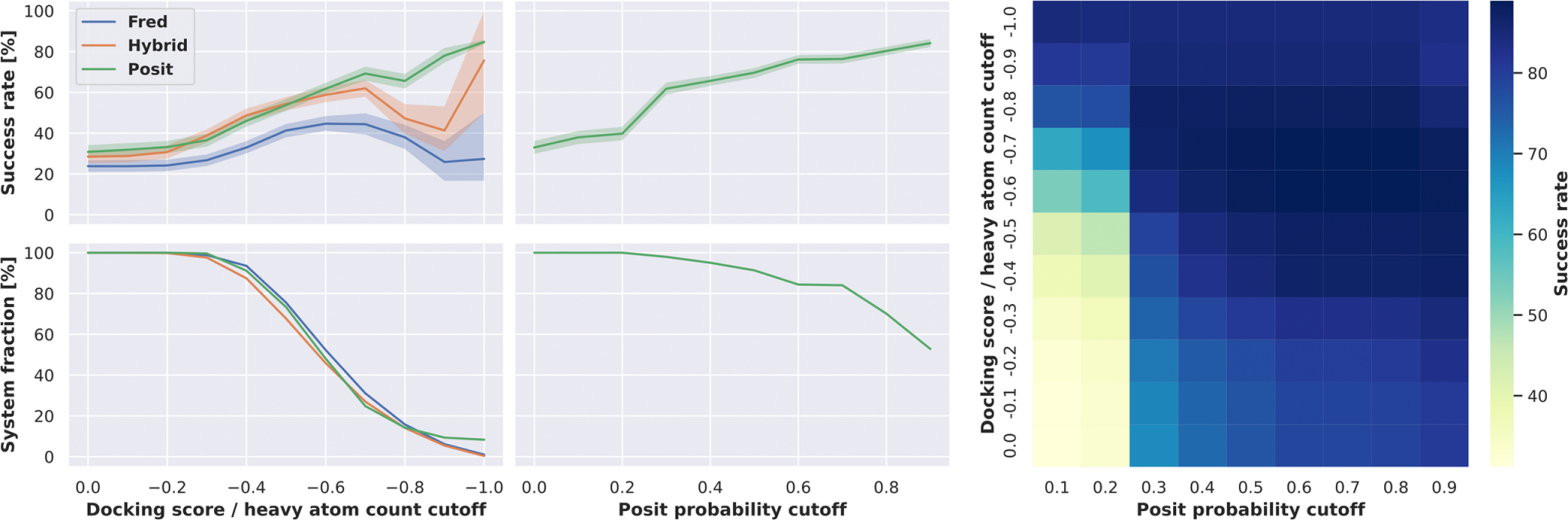
Docking success rates improve when applying more stringent docking scores or Posit probability cutoffs at the expense of suitable systems for docking. The docking success rate increases to more than 60% for Fred and Hybrid when only picking from docking poses with a heavy atom count-normalized docking score equal to or lower than −0.6. However, this also excludes 50% of the benchmark structures, for which no docking poses were generated satisfying this cutoff. In contrast, a Posit probability cutoff of 0.3 also allows for a docking success rate of 60% but excludes only less than 5% of the benchmark systems. Reported success rates and confidence intervals were estimated using bootstrapping. The heat map shows that docking poses with a Posit probability below 0.3 can have a docking success rate of more than 60% if the heavy atom count-normalized docking score is −0.8 or lower. Another version of this figure without normalization of the docking score is available in the appendix (Figure S4), but the overall findings do not change.

We observed an increasing overall success rate upon application of a more stringent docking score cutoff (Figure 4). This trend reverts for Hybrid and Fred when surpassing a heavy atom count normalized docking score cutoff of −0.8. However, there are less than 20% of the systems left at this cutoff, which is also reflected in an increased confidence interval. Additionally, we observed a relatively large fraction of ATP analogues in the remaining systems at such high cutoffs, which may add an artificial bias to the docking performance. For example, the phosphate groups of the ATP analogues have multiple charged centers, which can perform strong charged interactions and consequently result in good docking scores. Contrary, the phosphates of the ATP analogues are very flexible and thus might be relatively hard to position correctly for a low RMSD docking pose.

Applying more stringent Posit probability cutoffs also excluded several systems from analysis, for which no sufficient docking pose could be generated. But in comparison to the normalized docking score, this affected far fewer systems. For example, at a Posit probability cutoff of 0.9, which is very close to the theoretical maximum of 1, 50% of the systems remained for analysis and showed a success rate of over 80% in generating low RMSD docking poses.

Overall, the Posit probability was found to be more successful in selecting low RMSD docking poses than that with the normalized docking score. However, the normalized docking score was found especially valuable for docking poses with a Posit probability below 0.3 (Figure 4, right). Here, a normalized docking score below −0.7 could reliably predict low RMSD docking poses. This finding suggests that both metrics could also be combined to pick low RMSD docking poses.

**Figure 5:**
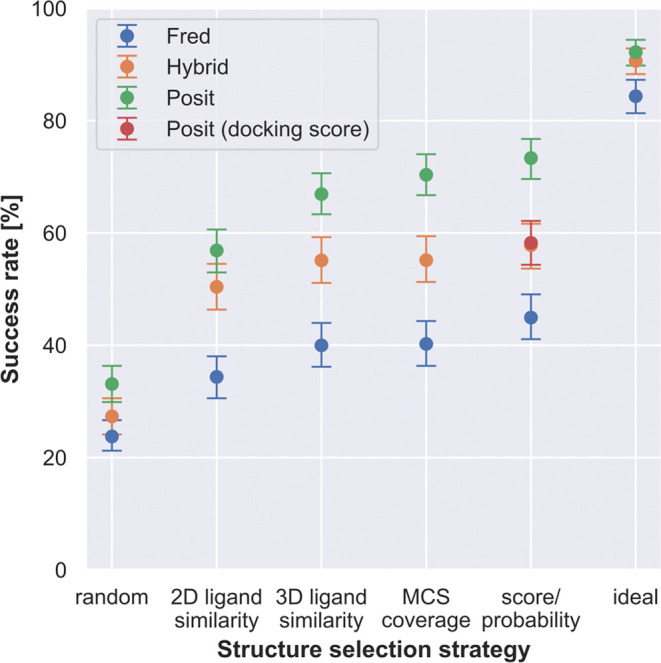
Kinase inhibitors pose recovery in cross-docking benefits from docking to multiple structures, selection of optimal ligand templates, and leveraging ligand information in an adaptive fashion (Posit). Posit clearly outperforms Hybrid and Fred. Success rates (pose below 2 Å) are over 589 kinase-inhibitor pairs. Kinase structure for docking randomly selected, by 2D or 3D ligand similarity to cocrystallized ligand, by MCS coverage, by docking score/Posit probability, or by identifying the lowest RMSD pose (ideal scenario). Reported success rates and confidence intervals were estimated using bootstrapping.

Next, we compared different docking methods and docking pose selection strategies on the full benchmark data set without applying any similarity or docking score cutoffs (Figure 5). Two extremes were included to help interpret the success rates. The **random** strategy randomly selects a kinase structure for docking for each system. In contrast, the **ideal** strategy reflects a situation in which we know which structure will deliver the lowest RMSD docking pose for each structure that is being reproduced. Both extremes show the expected range of success rates for different strategies, for which we should not perform worse than the random strategy but will also not perform better than the ideal strategy. The best combination of the docking method and selection strategy was the Posit algorithm selecting the best docking pose according to the Posit probability (73.3% success rate). However, this was not statistically significantly better than picking the X-ray structure for docking with the most similar cocrystallized ligand according to 3D similarity reflecting shape and electrostatics (66.9% success rate) or with the highest MCS coverage (70.4% success rate). This is important because selecting a structure based on Posit probability requires docking into all structures, while the 3D similarity and MCS coverage to the cocrystallized can be calculated beforehand. A promising option is available in the OpenEye Toolkits,^35^ in which the user can provide multiple protein structures for docking with Posit, and an automated routine will pick the most suitable protein structure for docking based on 2D and 3D similarity between cocrystallized ligand and small molecule to dock. This option was not included in this study since we were interested in receiving docking poses for all structures.

Overall, Posit was found to be the most successful docking method in reproducing binding poses from X-ray structures (Figures 2 and 5). However, Posit also generated more poses with a higher (more unfavorable) docking score than that of Fred and Hybrid (Figure S5), which indicates steric clashes or high strain ligand conformations. This is likely caused by a strong bias from the cocrystallized ligand when placing the new ligand in the binding site. An energy minimization or short molecular dynamics simulation could potentially relax such poses. A pose relaxation option is available in the OpenEye Toolkits,^35^ but this has not been tested in this study, since we wanted to focus this study on the docking performance and not different force field behaviors.

### Transferring Ligands for Posit Docking Does Not Improve Performance

After discovering the good performance of Posit, we implemented and tested a customized docking protocol employing Posit with a transferred cocrystallized ligand template. In this protocol, the KLIFS database is searched for similar cocrystallized ligands according to shape and electrostatics compared to the ligand that should be docked. The search is thereby covering all available kinase structures limited to the same DFG and αC helix conformation. The structure with the most similar ligand is then superimposed using functionality from the OpenEye Toolkits^35^ to the structure the ligand should be docked into, and the new, more similar ligand is transferred. This procedure was thought to improve the performance of the Posit method, since it is highly dependent on the similarity to the cocrystallized ligand (Figure 3).

**Figure 6:**
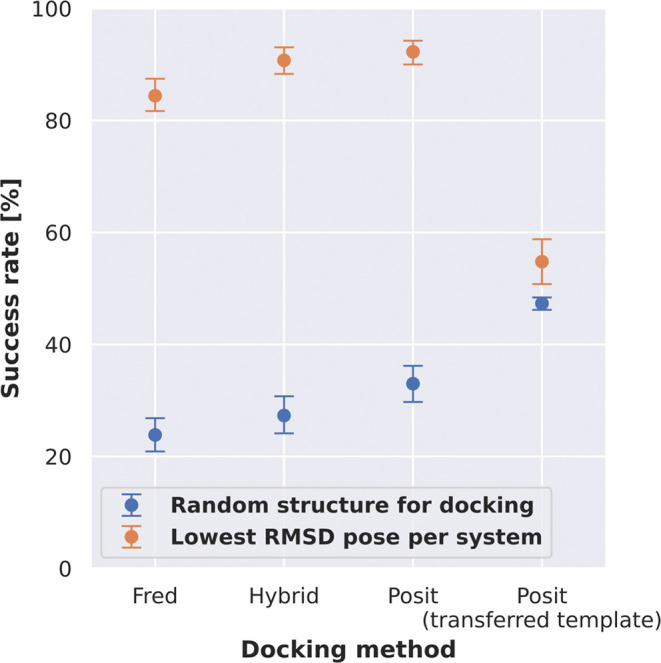
Transferring more similar ligands from other kinase structures improves the success rate for Posit when docking into a single kinase structure but not when docking into multiple kinase structures. A customized docking protocol was implemented employing Posit with a transferred cocrystallized ligand template. The ligand cocrystallized to each structure was thereby replaced by the most similar ligand compared to the ligand to dock found in the KLIFS database according to shape and electrostatics.

As expected, the similarity of the ligand template used to bias the Posit method could be increased by transferring more similar ligands from other kinase structures (Figure S6). The success rate for randomly picked kinase structures for docking increases from 33.0 to 47.3% when transferring a more similar template ligand from another kinase structure (Figure 6). However, the success rate for the lowest RMSD pose generated per system decreases from 92.2 to 54.8%. Additionally, the success rate of the lowest RMSD pose per system (54.8%) is worse than docking into structures with the most similar cocrystallized ligand according to 3D similarity without applying the transfer protocol (66.9%, Figure 5). This shows that transferring more similar ligands from other kinases does not always improve the docking performance. However, ligand transfer was also performed for very distantly related kinases, e.g., ligands from atypical kinases were transferred into structures of tyrosine kinases. Restricting the ligand transfer to be performed only with a kinase group may improve the performance, but it was not tested in this study.

### Posit Probability Can Discriminate Different Kinase Conformations with Larger Structural Rearrangements

The benchmark data set contains two kinases crystallized in different activation loop conformations, i.e., human AurA and human BRAF (Table 1). To assess whether molecular docking could also identify the correct kinase conformation, another experiment was performed by docking ligands into kinase structures with different DFG or αC helix conformations. Here, we wanted to assess (i) if the most successful docking strategy, Posit, with picking a docking pose via posit probability (Figure 5), can identify the kinase conformation it was cocrystallized with and (ii) if including multiple conformations has an effect on the overall docking performance.

**Figure 7:**
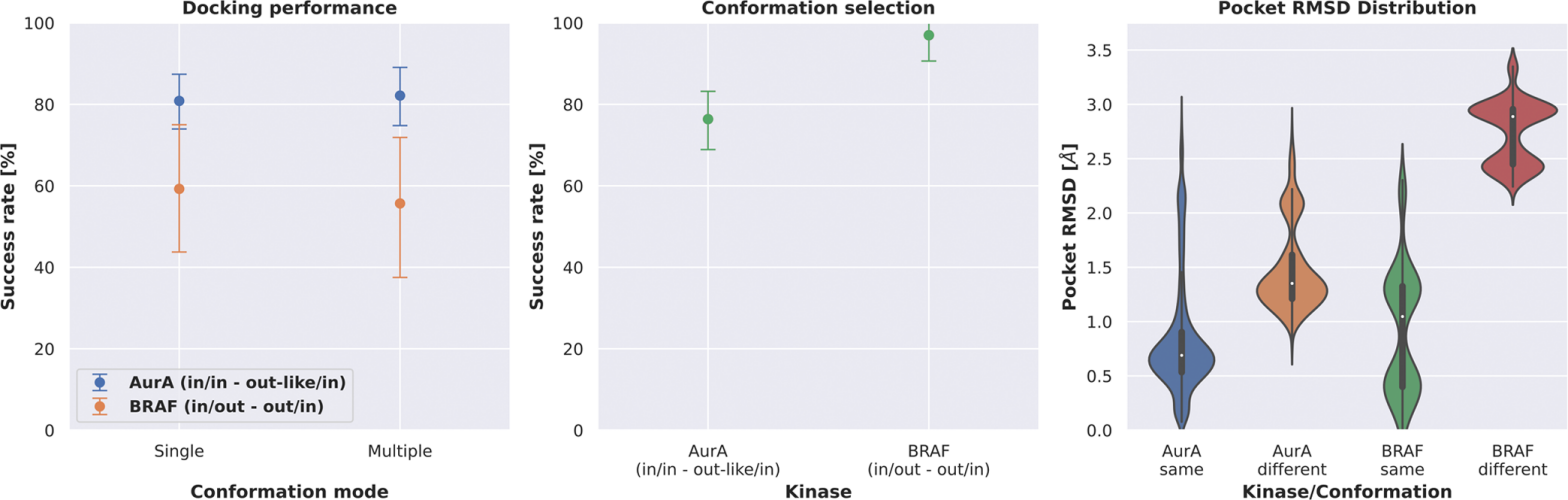
Posit docking performance is not significantly affected by docking into kinase structures of different conformations. The Posit probability can pick the correct kinase conformation in the majority of the included structures. The docking benchmark set contains structures for two kinases in two different conformations (DFG/αC helix motifs), i.e., AurA: in/in vs out-like/in and BRAF: out/in vs in/out (Table 1). Left: The docking success rate is not statistically different when selecting the docking pose with the best Posit probability from docking poses generated by docking into structures with the correct conformation or two different conformations. Middle: Detecting the correct kinase conformation with the Posit probability is more successful for larger structural differences, i.e., out/in vs in/out compared to in/in vs out-like/in. Right: The larger structural rearrangement in the case of BRAF (in/out vs out/in) compared to AurA (in/in vs out-like/in) is reflected in the pocket RMSD, which rationalizes the better conformation selection success rate for the BRAF kinase.

The conformational changes for both AurA and BRAF were first analyzed in terms of Pocket RMSD calculated for the 85 KLIFS binding site residues (Figure 7 right).^28^ According to the RMSD, the conformation change observed in the case of BRAF (out/in vs in/out) is larger compared to AurA (in/in-out-like/in).

The human AurA structures included in this benchmark data set were crystallized in the DFG in/αC helix in the conformation and the DFG out-like/αC helix in the conformation, which represents a relatively small conformational change. The docking success rate did not significantly differ when comparing the results of docking into structures of the same kinase conformation only or in structures of both kinase conformations (Figure 7, left). Also, the structure with the best Posit probability was in the majority (76.4%) of docked ligands in the correct kinase conformation (Figure 7, middle).

Human BRAF was crystallized in the DFG in/αC helix out conformation and the DFG out/αC helix in conformation, which represents a rather large conformational change. Again, the docking success rate did not differ significantly when comparing the results of docking into structures of the respective kinase conformation only or in structures of both kinase conformations (Figure 7, left). However, compared to the relatively small conformational change in the case of human AurA, the Posit method was able to select the correct kinase conformation for almost all docked ligands (96.9%, Figure 7, middle).

These results show that Posit and potentially other docking tools are more likely to identify the correct kinase conformation in the case of larger structural rearrangements. However, due to the flexible nature of kinases, it is possible that structures selected by docking that our experimental approach annotates as putative incorrect kinase conformations (in the case of human AurA) may actually represent a viable bound conformation. Such structure may simply have not been observed crystallographically due to crystallization conditions (Table 2) or crystal packing artifacts.^38^ In aqueous solutions, more than one conformation may be important for ligand binding even if not observed in an X-ray structure.^34^

## Conclusions

Employing protein–ligand complex information for predicting bioactivity with ML models holds significant potential to improve performance.^7,10,11^ Combining such models with highly automated and accurate docking pipelines is both critical for predicting the bioactivity of newly designed compounds and can be useful for enlarging the data set available for training by providing structures for training data for which only activities are available.^39^

In this study, we presented a cross-docking benchmark for highly automated docking pipelines integrated into the KinoML framework. The benchmark data set focuses on protein kinases and was carefully curated with regard to the flexible nature of kinases and comprises 589 protein kinases cocrystallized with 423 different ATP-competitive ligands.

We found that the investigated docking methods Fred, Hybrid, and Posit from the OpenEye Toolkits^35^ were able to recover the majority of ligand poses observed in the experimentally resolved structures. However, the docking performance was dependent on the docking method and the selected kinase structure for docking. Methods biased by the cocrystallized ligand (Hybrid and Posit) were more successful than the standard docking method Fred. Docking into a kinase structure with a more similar cocrystallized ligand was found to positively impact docking performance, which is in line with previous cross-docking studies.^20−22^

Also, better docking scores and Posit probabilities indicated lower RMSD docking poses. However, such an approach requires docking into multiple structures, whereas the ligand similarity to the cocrystallized ligand can be calculated before performing the more computationally expensive docking calculation. In addition, docking into multiple structures strongly increased the chance of generating a low RMSD docking pose for all investigated docking methods, e.g., from 33.1% when docking into one random structure with Posit to 83.2% when docking into 20 random structures (Figure 2 right). Interestingly, the docking performance of the most successful docking method, Posit, was not affected by docking into different kinase conformations. And the Posit probability was able to predict the correct kinase conformation in most tested kinase systems.

Considering the improved success rates of docking algorithms employing information on a cocrystallized ligand such as shape or maximum common substructure, we would like to strongly encourage other docking software developers to include these routines. At the time of writing this manuscript, we were not aware of open-source docking tools allowing to bias docking results by the shape of a cocrystallized ligand.

This benchmark was performed not only to test the designed docking pipelines but also to learn for future structure-informed machine learning experiments for bioactivity prediction. Based on our findings, the Posit docking method was the most successful to generate docking poses with the lowest RMSD when docking into the kinase structure with the most similar cocrystallized ligand. If the downstream ML model can handle multiple input docking poses for a single kinase-inhibitor pair, then the model could learn the relevance for each input docking pose based on ligand similarity and docking score. Also, the ML model is more likely to see a low RMSD docking pose when providing poses from docking into multiple structures rather than multiple poses from docking into the same structure.

Finally, it would be interesting to see the performance of the newly developed ML-based docking approaches in concert with bioactivity predictions for this benchmark data set.^12−14,16^ However, this experiment was not included in this study, since the overlap of the training set of these ML-based docking approaches with our benchmark data set would complicate interpretation of the results. In one of our recent publications, we used the template docking introduced in this benchmark to generate over 120k kinase-ligand complexes.^40^ The study demonstrates that using synthetic binding poses can significantly enhance the predictive precision of machine learning models, here an E(3)GNN.

## Detailed Methods

### Benchmark Data Set

The OpenCADD-KLIFS module was used to query for available kinase structures in the PDB in January 2022 (Table 3).^26,27,29^ The retrieved 12,572 KLIFS structures were filtered for the presence of a single ligand in the ATP-binding site, since the docking methods Hybrid and Posit require a cocrystallized ligand for pocket definition and the presence of multiple ligands in the same binding pocket likely hinders correct sampling of the docking programs. For each PDB entry, only the highest quality chain was retained according to the structure quality reported by KLIFS, leading to 5,017 remaining entries. Next, KLIFS entries were excluded that contain mutations in the 85 KLIFS residues defining the binding site as well as KLIFS entries with an incompletely resolved ATP-binding site. This step should ensure that modeling of missing or mutated residues does not bias the docking benchmark and left 1,914 KLIFS entries for further processing. The remaining entries were filtered for a reasonable molecular weight (150–1000 Da) of the cocrystallized ligand and for SMILES representation stored in the PDB interpretable by RDKit and OpenEye Toolkits^35^ that are later used for docking and analysis. For example, PDB entry 5C01 does not have a valid SMILES representation stored in the PDB. This step removed 27 KLIFS entries. Next, structures were removed with multiple instances of the same ligand bound to the same protein chain, removing another 27 KLIFS entries. The remaining 1,888 KLIFS entries were grouped to select those kinases that have at least 10 different KLIFS entries available. Finally, the most populated kinases were picked for each kinase group (TK, TKL, STE, ···) and conformation (DFG/αC helix in/in, in/out, out-like/in and out/in) to ensure covering different kinase groups and conformations leaving the final benchmark data set comprising 589 KLIFS entries cocrystallized with 423 different ligands (Table 3).

**Table 3:** Benchmark Data Set Creation^a^

selection step	number of remaining KLIFS entries
all available KLIFS entries	12,572
single orthosteric ligand	10,439
highest quality chain per PDB structure	5017
no alterations or unresolved residues in the KLIFS binding site	1942
no ligands with unreasonable molecular weight or not handled by RDKit or OpenEye Toolkits	1915
exclude entries with multiple instances of the same ligand bound to the same protein chain	1888
at least 10 available structures per kinase and conformation from different kinase groups and conformations	589

a Sequential selection steps reduce the number of available KLIFS entries for docking from 12,572 to 589.

### Molecular Docking

Protein structure preparation was performed using OEChem and Spruce functionality from the OpenEye Toolkits 2021.1.1^35^ implemented into the KinoML framework. Missing side chains were built using the Spruce toolkit, and all protein chain termini were capped with NME and ACE residues. The resulting protein–ligand complex was protonated at pH 7.4 using the OEChem toolkit.

Molecular docking was performed using OEDocking, Omega, and Quacpac functionality from the OpenEye Toolkits 2021.1.1^35^ implemented into the KinoML framework.

For each molecule to dock, reasonable tautomers at pH 7.4 were determined using Quacpac. Undefined stereo centers were enumerated with Omega. Finally, up to 800 conformations were generated for each enantiomer and tautomer using Omega with sampling in the Pose mode.

For the Fred and Hybrid docking algorithms, docking was performed with a high search resolution. The 5 best docking poses, according to the Chemgauss4 docking score, were returned and written to disk. Posit docking calculations were performed with the IgnoreNitrogenStereo option set to True, which is the recommended setting according to the documentation. The pose relaxation was turned off, and the posed molecule subsequently scored with the Chemgauss4 docking score.

### Docking Pose Analysis

For the calculation of the heavy atom RMSD of a docking pose to its reference X-ray structure, a structural superposition was performed to align kinase binding site residues, as described below. This step involved Spruce functionality from the OpenEye Toolkits 2021.1.1^35^ implemented into the KinoML framework. The superposition was thereby restricted to the heavy atoms of the 85 KLIFS residues retrieved via the OpenCADD-KLIFS module.^27,29^ Subsequently, the heavy atom RMSD of cocrystallized ligand and docking pose was calculated using SpyRMSD.^41^

The 2D similarity between the small molecule to be docked and the cocrystallized ligand was calculated with Morgan fingerprints with features, radius of 2 and 2,048 bits implemented in the RDKit library. The maximum common substructure (MCS) coverage was calculated with the RDKit library. The number of MCS atoms between two molecules was divided by the number of heavy atoms of the ligand being docked to generate the MCS coverage. The 3D similarity in the form of shape and electrostatics was determined for enumerated conformations of the small molecule to dock to the 3D structure of the cocrystallized ligand. The overlay was performed using Shape, Omega, and Quacpac functionality from the OpenEye Toolkits 2021.1.1^35^ implemented into the KinoML framework. For each molecule, reasonable tautomers at pH 7.4 were determined using Quacpac. Undefined stereo centers were enumerated with Omega. Finally, up to 200 conformations were generated for each enantiomer and tautomer using Omega with sampling in the Classic mode. These conformations were overlaid to the 3D structure of the cocrystallized ligand, and the 3D similarity was determined using the TanimotoCombo score considering shape and electrostatics.

### Statistical Measures

Per experiment, ∼ 40,000 docking runs were performed in this cross-docking study, docking each ligand into each structure of the same kinase and conformation. To estimate the impact of, e.g., ligand similarity or docking score on docking performance, the success rates, including 95% confidence intervals, were calculated using 1000 bootstrap samples.

For example, the reported success rate of 33.0% for Posit when docking a ligand into a randomly selected structure of the same kinase and conformation (Figure 2) was generated by (i) randomly picking a single docking pose for each ligand, (ii) calculating the mean success rate over all structures, (iii) performing steps i and ii 1000 times, and (iv) calculating the mean success rate over all bootstrap samples as well as the 95% confidence interval.

The detailed procedure for each analysis can be found in the Jupyter notebooks of the accompanying kinase-docking-benchmark repository.

## Data Availability

The kinase-docking-benchmark repository is made publicly available and stores all relevant Python scripts and Jupyter notebooks to reproduce the presented docking benchmark results and analysis. The associated docking pipelines are available in the KinoML framework. The generated docking poses are stored in an OSF project.

## Supporting Information Available

Additional figures (Figures S1–S6) mentioned in the text for a more comprehensive understanding of the chemical space covered by the benchmark data set and more details on the benchmark results (PDF)
The docking benchmark data set (XLSX)
