# Supplementary material for: Benchmarking Cross-Docking Strategies in Kinase Drug Discovery
Source: J Chem Inf Model. 2024 Nov 19;64(23):8848–58. doi: 10.1021/acs.jcim.4c00905 (PMC11661510; doi:10.1021/acs.jcim.4c00905)
Supplement: Supplementary file 1 — ci4c00905_si_001.pdf [file ci4c00905_si_001.pdf]

Supporting Information

Benchmarking Cross-Docking Strategies in Kinase Drug Discovery

David A. Schaller[1,2], Clara D[3]. Christ, John D. Chodera[2,], Andrea Volkamer[1,4,]**

[1] In Silico Toxicology and Structural Bioinformatics, Institute of Physiology, Charité – Universitätsmedizin Berlin, corporate member of Freie Universität Berlin and Humboldt-Universität zu Berlin, Augustenburger Platz 1, 13353 Berlin, Germany

[2] Computational and Systems Biology Program, Sloan Kettering Institute, Memorial Sloan Kettering Cancer Center, New York, NY 10065, USA

[3] Molecular Design, Research and Development, Pharmaceuticals, Bayer AG, 13342 Berlin, Germany

[4] Data Driven Drug Design, Faculty of Mathematics and Computer Sciences, Saarland University, Saarbrücken, Germany

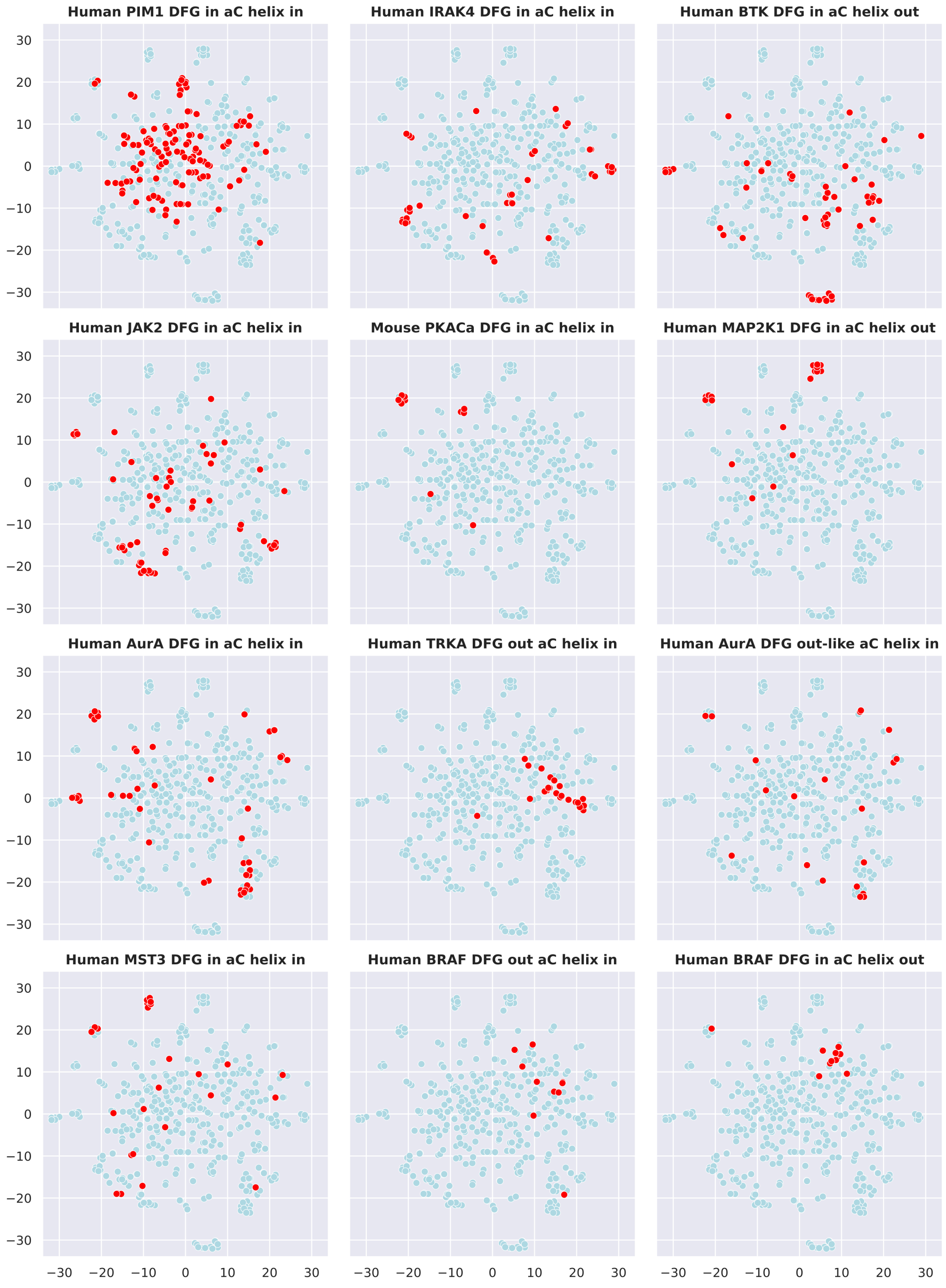

Figure S1. Chemical space of co-crystallized ligands for each kinase and conformation included in the docking benchmark data set. The chemical space is visualized via T-SNE representation of 50 principal components accounting for 64 % of observed variance in the principal component analysis. Blue dots represent ligand data points of the entire data set, red dots of the respective kinase and conformation. The principal components are based on Morgan fingerprint with features of radius 2 and 2,048 bits implemented in the RDKit. The principal components analysis and T-SNE representation was performed with Scikit-learn.[1]

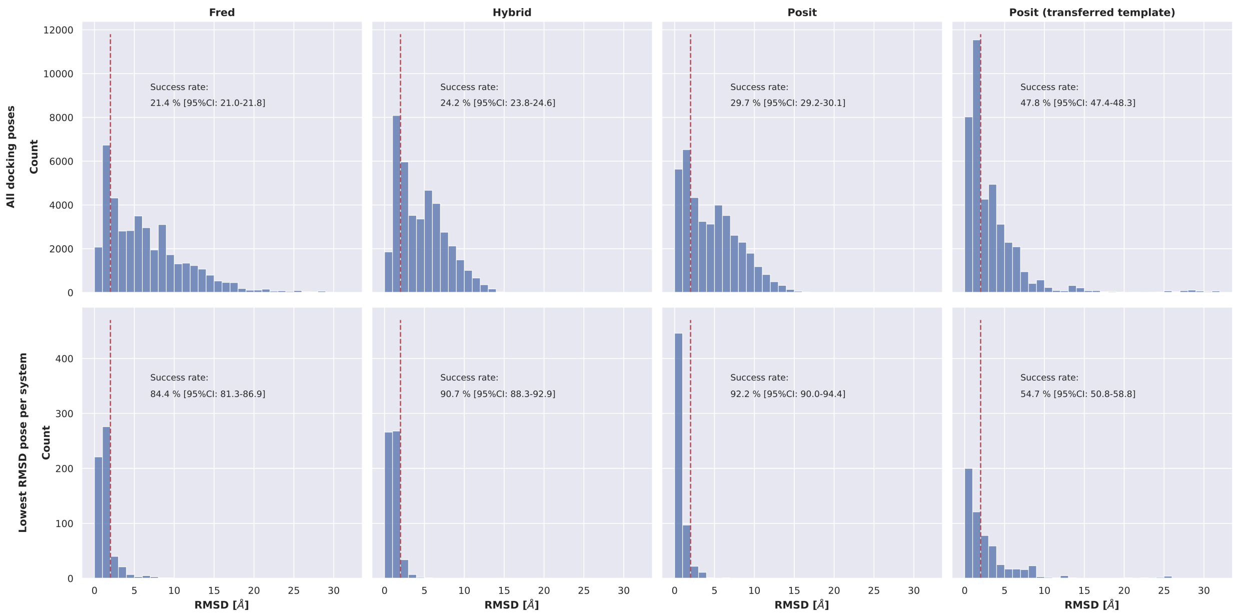

Figure S2. Assessment of the accuracy of kinase inhibitor pose recovery from cross-docking into kinase structures in the same conformation. The benchmark results show that low RMSD docking poses can be generated for the majority of the systems; docking into multiple structures is important. Top row: Average success rates (pose with RMSD below 2 Å) for all ~40K docking poses. Bottom row: Lowest RMSD poses for 589 kinase:inhibitor pairs in total. Reported success rates and confidence intervals were estimated using bootstrapping.

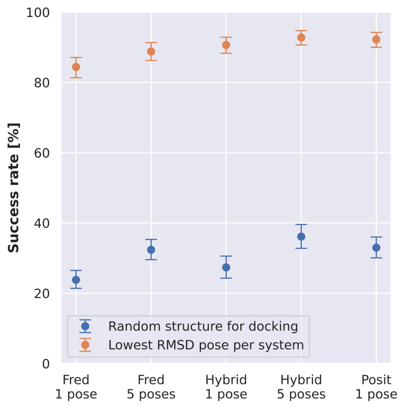

Figure S3. Returning multiple docked poses does significantly improve the success rate of generating a low RMSD docking pose when docking into a single randomly chosen kinase structure as indicated by the non-overlapping confidence intervals (error bars in attached to dots) in blue for Fred and Hybrid. When docking into all available structures returning multiple poses does not significantly improve the best attainable success rate as indicated by the overlapping confidence intervals in orange for Fred and Hybrid. Posit was not included since the used OpenEye Toolkits (2021.1.1) version did not support the generation of multiple poses. Reported success rates and confidence intervals were estimated using bootstrapping.

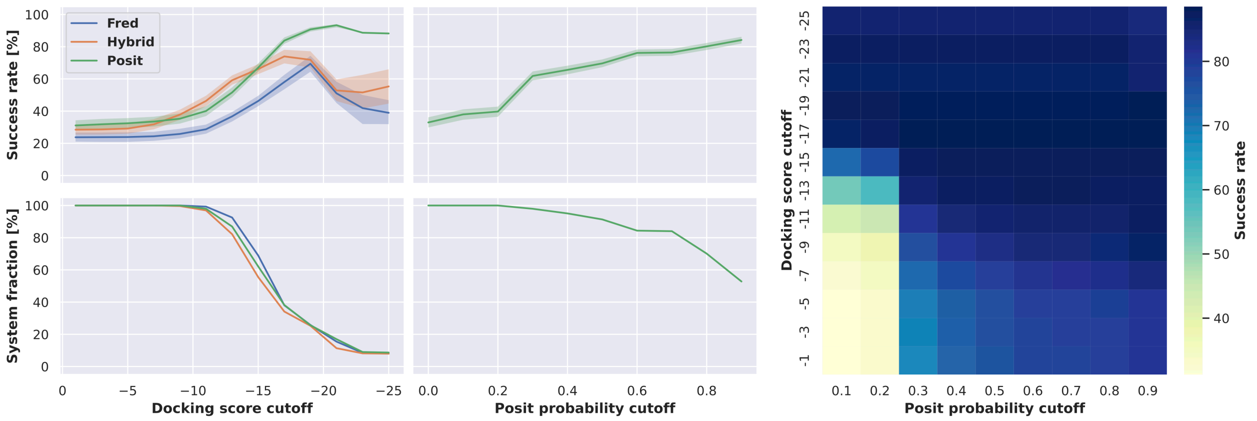

Figure S4. Docking success rates improve when applying more stringent docking score or Posit probability cutoffs. Figure version without normalization of docking score by number of heavy atoms, compare to **Figure 4** in the manuscript. Reported success rates and confidence intervals were estimated using bootstrapping.

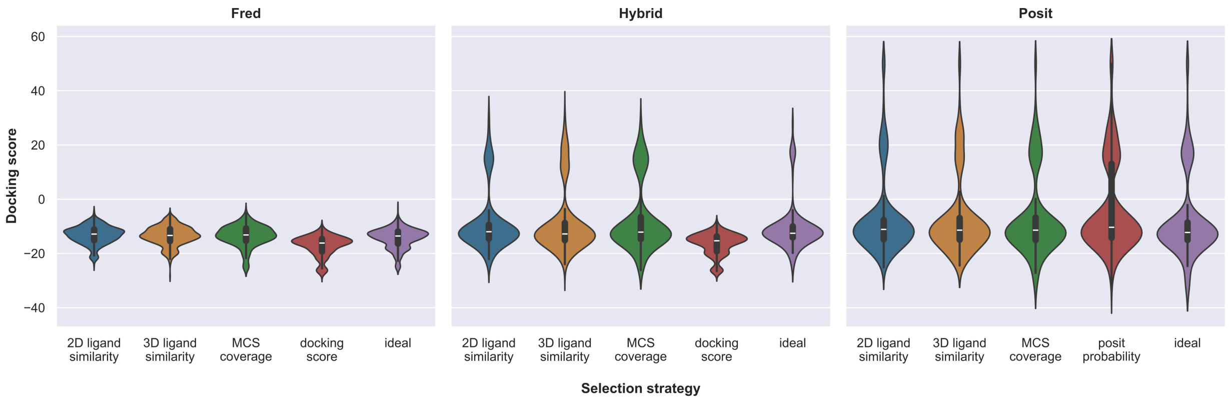

Figure S5. Posit and Hybrid generate more docking poses with high docking score compared to Fred. Poses selected by 2D or 3D ligand similarity to the co-crystallized ligand, by maximum common scaffold (MCS), by docking score / Posit probability or by identifying the lowest RMSD pose (ideal scenario) for different docking methods. An upper bound of 50 was introduced for the docking scores to allow proper visualization. Docking with the Fred method and selecting docking poses via the docking scores generates docking score distributions with the lowest docking scores. Docking with Posit generates several high docking score poses that indicate atom clashes which may require additional structural optimization.

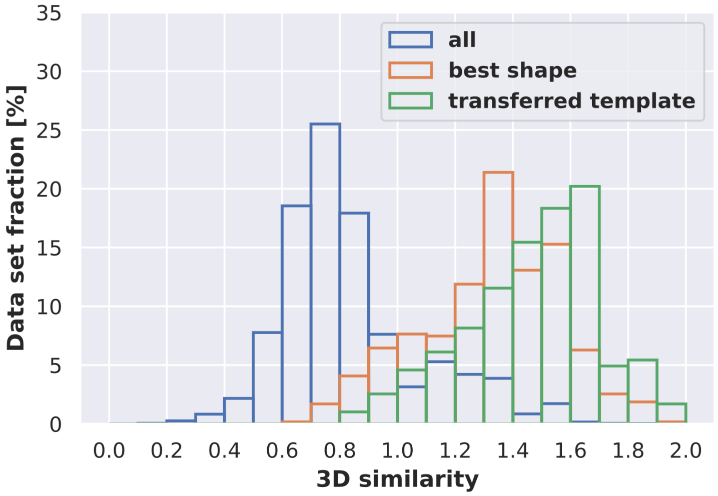

Figure S6. Comparison of Posit ligand template similarity for different docking experiments. When considering all ~40K docking runs, the average ligand similarity according to shape and electrostatics between co-crystallized ligand and molecule to dock was below 1. Restricting this analysis to the structure with the most similar co-crystallized ligand increased the average 3D similarity, which has a beneficial effect on the docking performance for all tested docking methods (**Figure 5**). This 3D similarity to the co-crystallized ligands can be even more increased when transferring more similar ligands from other kinases. However, this strategy only was found to improve performance when docking into a single randomly selected kinase structure (**Figure 6**).

References

(1) Pedregosa, F.; Varoquaux, G.; Gramfort, A.; Michel, V.; Thirion, B.; Grisel, O.; Blondel, M.; Müller, A.; Nothman, J.; Louppe, G.; Prettenhofer, P.; Weiss, R.; Dubourg, V.; Vanderplas, J.; Passos, A.; Cournapeau, D.; Brucher, M.; Perrot, M.; Duchesnay, É. Scikit-Learn: Machine Learning in Python. **2012**. https://doi.org/10.48550/ARXIV.1201.0490.
